# Transcriptomic analysis of the biosynthesis, recycling, and distribution of ascorbic acid during leaf development in tea plant (*Camellia sinensis* (L.) O. Kuntze)

**DOI:** 10.1038/srep46212

**Published:** 2017-04-10

**Authors:** Hui Li, Wei Huang, Guang-Long Wang, Wen-Li Wang, Xin Cui, Jing Zhuang

**Affiliations:** 1 Tea Science Research Institute, College of Horticulture, Nanjing Agricultural University, Nanjing 210095, China; 2 State Key Laboratory of Crop Genetics and Germplasm Enhancement, College of Horticulture, Nanjing Agricultural University, Nanjing 210095, China

## Abstract

Ascorbic acid (AsA), known as vitamin C, is an essential nutrient for humans and mainly absorbed from food. Tea plant (*Camellia sinensis* (L.) O. Kuntze) leaves can be a dietary source of AsA for humans. However, experimental evidence on the biosynthesis, recycling pathway and distribution of AsA during leaf development in tea plants is unclear. To gain insight into the mechanism and distribution of AsA in the tea plant leaf, we identified 18 related genes involved in AsA biosynthesis and recycling pathway based on the transcriptome database of tea plants. Tea plant leaves were used as samples at different developmental stages. AsA contens in tea plant leaves at three developmental stages were measured by reversed-phase high-performance liquid chromatography (RP-HPLC). The correlations between expression levels of these genes and AsA contents during the development of tea plant leaves were discussed. Results indicated that the l-galactose pathway might be the primary pathway of AsA biosynthesis in tea plant leaves. *CsMDHAR* and *CsGGP* might play a regulatory role in AsA accumulation in the leaves of three cultivars of tea plants. These findings may provide a further glimpse to improve the AsA accumulation in tea plants and the commercial quality of tea.

The tea plant (*Camellia sinensis* (L.) O. Kuntze) is an important economic crop in China^1^. The leaves of tea plants have been classically recognized as a good source for producing tea including oolong tea, black tea, green tea and white tea. The production of tea was estimated at 1,939,457 tons in China in 2013 from the FAOSTAT website (http://faostat3.fao.org). Nowadays, tea is one of the most popular beverages in the world. Tea plants are rich in many nutritious compositions, such as theanine, caffeine, theobromine, theophylline, and ascorbic acid (AsA)^2^,^3^. Drinking tea may help reduce the risks of cancer^4^,^5^,^6^.

AsA is an enzyme cofactor in plants and an essential nutrient for humans. Furthermore, AsA possesses a series of observable physiologic functions for reducing the risks of scurvy, lung cancer, and cardiovascular disease^7^,^8^,^9^. l-Gulonolactone oxidase is essential for the synthesis of AsA; however, humans and other primates lack this enzyme^10^. Therefore, humans must absorb AsA from diet, such as vegetables and fruits which contain a rich concentration of AsA. AsA may improve catechins bioavailability by enhancing intestinal uptake from tea^11^. Exogenous AsA can increase the flavanol concentration by 20% in green tea^12^.

Based on previous evidence, four principal biosynthesis pathways of AsA were propounded in plants, namely, l-galactose (l-Gal) pathway, l-gulose pathway, d-galacturonate pathway, and *myo*-inositol pathway^13^,^14^,^15^,^16^. l-Gal was important for AsA biosynthesis in higher plants. The l-Gal pathway was predominant in all of the four pathways until now. The other alternative pathways also played a supporting role in the AsA biosynthesis in plants. Wheeler and his colleagues found that mannose and l-Gal were effective precursors for the biosynthesis of AsA, GDP-mannose 3,5-epimerase catalysed the transformation of mannose into l-Gal^13^. Nine enzymes are involved in the l-Gal pathway^17^, namely, phosphoglucose isomerase (PGI), phosphomannose isomerase (PMI)^18^, phosphomannose mutase (PMM)^19^, GDP-d-mannose-3′,5′-epimerase (GME)^20^, GDP-d-mannose pyrophosphorylase (GMP)^21^, GDP-l-galactose phosphorylase (GGP)^22^, l-galactose-1-P phosphatase (GPP)^23^; l-galactose dehydrogenase (GalDH)^24^, and l-galactono-1,4-lactone dehydrogenase (GalLDH)^25^. In the l-gulose pathway, GDP-d-mannose-3′,5′-epimerase (GME) converts GDP-D-mannose to GDP-l-galactose^15^. In the d-galacturonate pathway, d-galacturonate reductase (GalUR) catalysed the transformation of d-galacturonic acid (d-GalUA) into l-galactonic acid^14^. The expression level of the *GalUR* gene was correlated well with AsA accumulation in strawberry^14^,^26^. In the *myo*-inositol pathway, phosphatase played an important role in the produce of *myo*-inositol^27^,^28^,^29^. *Arabidopsis* lines overexpressing *miox4*, a key gene in the *myo*-inositol pathway, showed an obvious increase in the AsA content^16^. In addition, the enzymes involved in the AsA recycling pathway include ascorbate oxidase (AO)^30^, ascorbate peroxidase (APX)^31^, dehydroascorbate reductase (DHAR)^32^, glutathione reductase (GR)^33^, and monodehydroascorbate reductase (MDHAR)^34^. Different pivotal enzymes may lead to distinct changes in the AsA concentration in higher plants. Overexpressing a strawberry *GalUR* gene in *Arabidopsis* resulted in a two- to three-fold increase in AsA levels^14^. Both transgenic tobacco and maize plants hosting *DHAR* gene exhibited higher AsA levels in foliar and kernel^35^. Overexpression of an acerola *GMP* gene in tobacco, showed a two-fold increase in the ascorbate content^36^, whereas overexpression of the *MIOX4* gene caused a two- and three-fold increase in the ascorbate content in *Arabidopsis* leaves^16^.

Recent studies have demonstrated that the main biosynthesis pathway of AsA was the l-Gal pathway in apple fruits and leaves of different ages^37^,^38^. Considerable evidence indicated that the l-Gal pathway was a principal route for AsA biosynthesis in most plants. For instance, the l-Gal pathway was a predominant biosynthetic route of ascorbate in apple leaves^38^. Similarly, the l-Gal pathway was found to be the primary pathway of AsA accumulation in carrots and radish roots^17^,^39^. Meanwhile, l-Gal pathway played a predominant role in AsA biosynthesis in peel and pulp of *citrus* fruits^40^.

The tea plant samples of transcriptome sequencing included mid-leaf ‘Yunnanshilixiang’ (Tea_T1) from Yunnan province, small-leaf ‘Chawansanhao’ (Tea_T2) from Jiangsu province, large-leaf ‘Ruchengmaoyecha’ (Tea_T3) from Hunan province, and small-leaf ‘Anjibaicha’ (Tea_T4) from Zhejiang province. These four tea plant samples of transcriptome sequencing were significantly different, including environmental adaptation and leaf size. In the present research, ‘Anjibaicha’ was a kind of small-leaf tea plants. ‘Yingshuang’ was a kind of mid-leaf tea plants. ‘Huangjinya’ was a kind of small-leaf tea plants. The AsA contents were different among the three tea plant cultivars. Based on the different contents of AsA, the three tea plant cultivars (‘Huangjinya’, ‘Anjibaicha’, and ‘Yingshuang’) were used as suitable samples for this research, and were used as samples in gene expression analyses. The related genes that involved in the biosynthesis and recycling pathways of AsA were identified from the tea plant transcriptome database^41^. Twelve genes involved in AsA biosynthesis and six genes related to the AsA recycling pathways were selected. The AsA content in tea plant leaves at three developmental stages in ‘Yingshuang’, ‘Huangjinya’, and ‘Anjibaicha’ were recorded. Finally, we investigated the expression levels of AsA-related genes in the three tea plant cultivars. This study will provide useful information for exploring of improving the content of AsA in the tea plants.

## Results

### Growth analysis of leaves at three developmental stages in three tea plant cultivars

**Figure 1:**
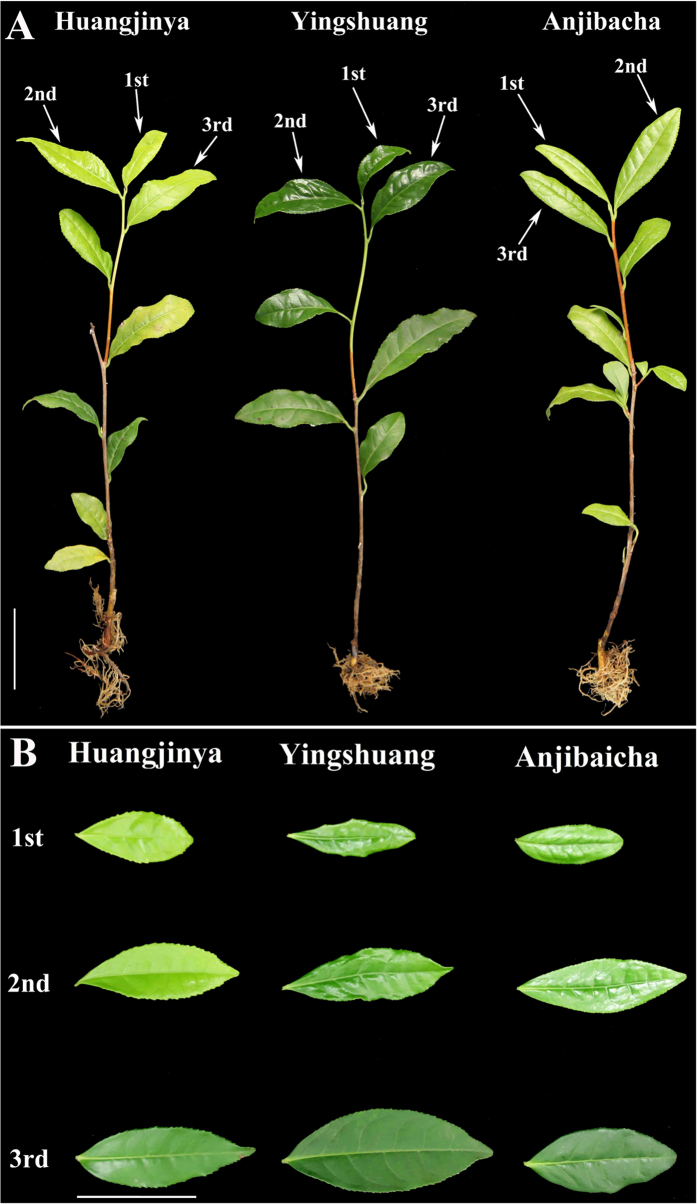
The three tea plant cultivars. (**A**) The plant of ‘Anjibaicha’, ‘Yingshuang’, and ‘Huangjinya’. (**B**) Tea plant leaves of three developmental stages: Stages 1 (1st leaf), 2 (2nd leaf) and 3 (3rd leaf). The lines beside the leaves represent 5 cm in that pixel.

The samples were sorted into three developmental stages, including stage 1 (1st leaf), stage 2 (2nd leaf), and stage 3 (3rd leaf) (Fig. 1). Three tea plants included ‘Anjibaicha’, ‘Yingshuang’, and ‘Huangjinya’.

### Changes in AsA content

**Figure 2:**
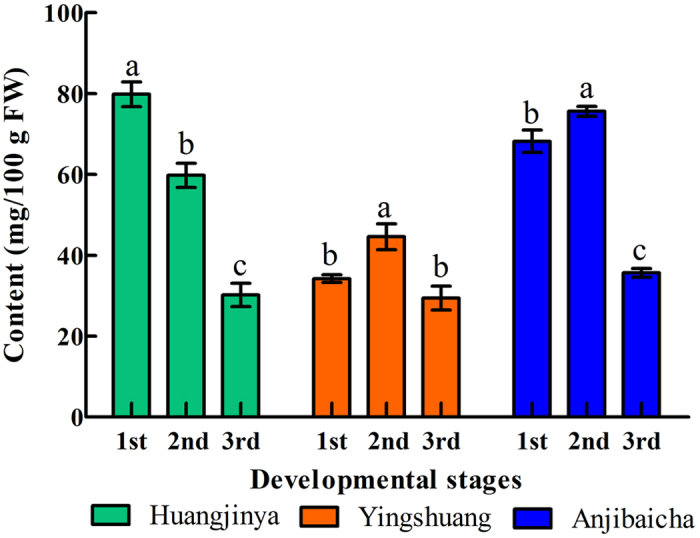
AsA contents of leaves at three developmental stages in three tea plant cultivars. Error bars represent standard deviation among three independent replicates. Data are means of three replicates ± SD.

**Figure 3:**
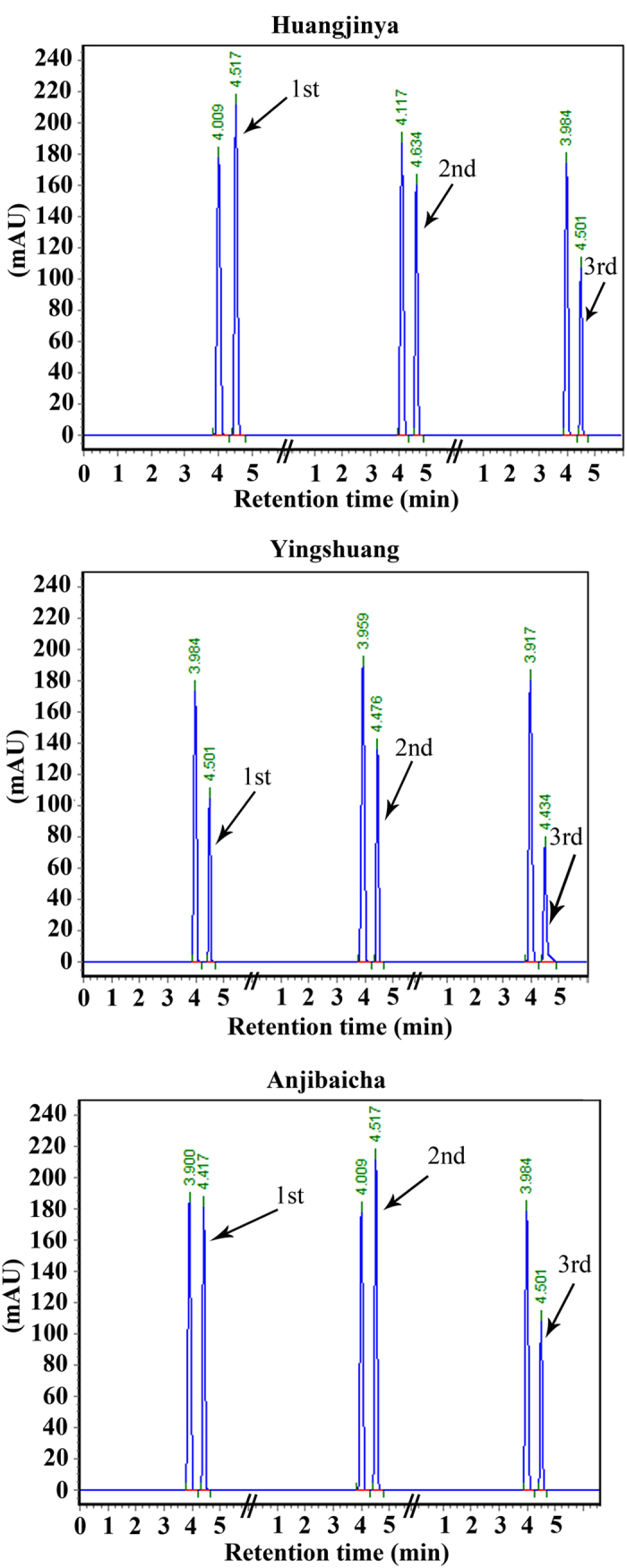
HPLC chromatogram of AsA in the leaves at three developmental stages from three tea plant cultivars.

The AsA content was measured at three leaf developmental stages in three tea plant cultivars by RP-HPLC (Figs 2 and 3). The highest concentration of AsA was detected at the first stage in ‘Huangjinya’ (79.81 mg/100 g FW), whereas the lowest content was observed at stage 3 in ‘Yingshuang’ (29.43 mg/100 g FW). The AsA content initially increased and then evidently decreased in ‘Yingshuang’ and ‘Anjibaicha’. A significant reduction of AsA content was observed during leaf development in ‘Huangjinya’.

### Expression levels of the genes involved in AsA biosynthesis in tea plants

**Figure 4:**
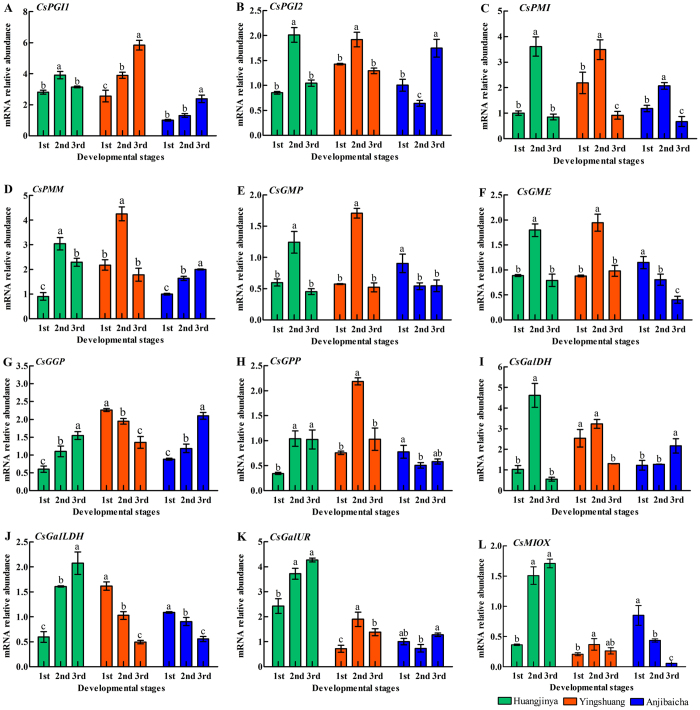
Expression level analyses of genes involved in AsA biosynthesis pathway in tea plant leaves. Genes involved in AsA biosynthesis pathway (**A**) Phosphoglucose isomerase (*CsPGI1*), (**B**) (*CsPGI2*), (**C**) phosphomannose isomerase (*CsPMI*), (**D**) phosphomannose mutase (*CsPMM*), (**E**) GDP-d-mannose pyrophosphorylase (*CsGMP*), (**F**) GDP-d-mannose-3′,5′-epimerase (*CsGME*), (**G**) GDP-l-galactose phosphorylase (*CsGGP*), (**H**) l-galactose-1-P phosphatase (*CsGPP*), (**I**) l-galactose dehydrogenase (*CsGalDH*), (**J**) l-galactono-1,4-lactone dehydrogenase (*CsGalLDH*). Error bars represent standard deviation among three qRT-PCR reaction replicates. Data are means of three replicates ± SD.

The expression levels of 12 genes involved in AsA biosynthesis were detected in leaves at different developmental stages of three tea plant cultivars by qRT-PCR (Fig. 4). The expression level of *CsPGI1* showed an upward trend in both ‘Anjibaicha’ and ‘Yingshuang’ during three developmental stages (Fig. 4A). The expression level of *CsPGI2* peaked at stage 2 then declined in ‘Huangjinya’ and ‘Yingshuang’. By contrast, the transcription level of *CsPGI2* decreased at the stage 2 and then increased in ‘Anjibaicha’ (Fig. 4B). *CsGalLDH* displayed a continuous decrease at three developmental stages in both ‘Anjibaicha’ and ‘Yingshuang’ (Fig. 4J). The expression levels of *CsGGP* and *GalLDH* experienced a similar upward trend in ‘Huangjinya’ (Fig. 4G,J).

### Expression levels of the genes involved in AsA recycling in tea plant

**Figure 5:**
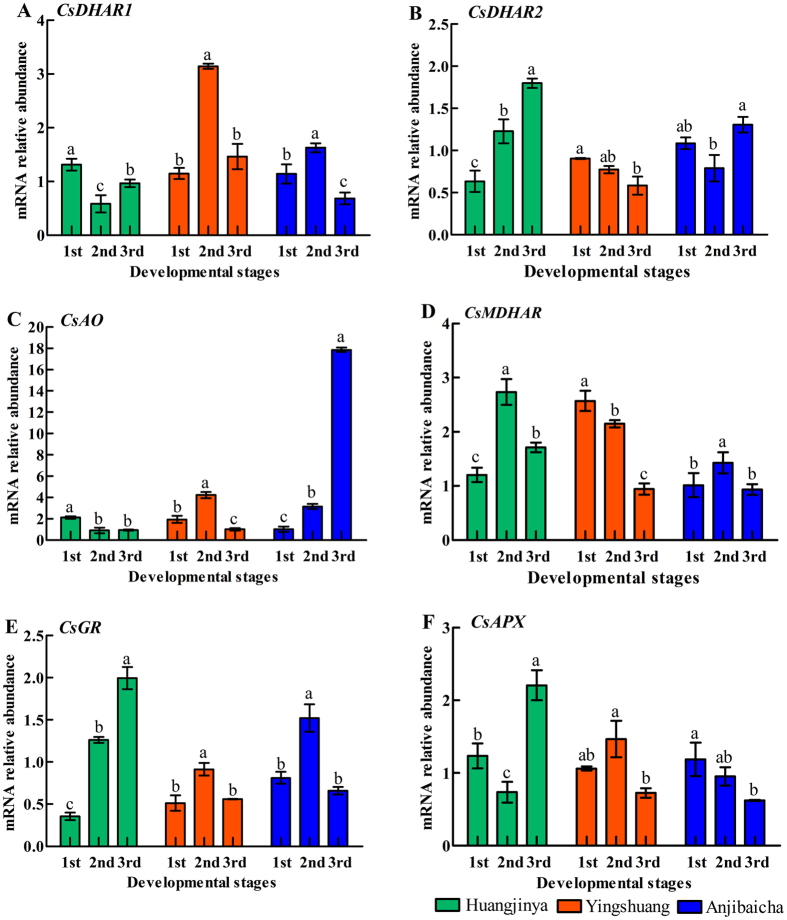
Expression level analyses of genes involved in AsA recycling pathway in tea plant leaves. Genes involved in recycling pathway include (**A**) dehydroascorbate reductase (*CsDHAR1*), (**B**) (*CsDHAR2*), (**C**) ascorbate oxidase (*CsAO*), (**D**) monodehydroascorbate reductase (*CsMDHAR*), (**E**) glutathione reductase (*CsGR*), (**F**) ascorbate peroxidase (*CsAPX*). Error bars represent standard deviation among three qRT-PCR reaction replicates. Data are means of three replicates ± SD.

The expression levels of six genes involved in the AsA recycling pathway were also detected in tea plants (Fig. 5). The expression levels of *CsDHAR1, CsMDHAR* and *CsGR* initially increased, followed by a decrease at the last stage in ‘Anjibaicha’ (Fig. 5A,D and E). The expression levels of *CsDHAR2* and *CsMDHAR* showed a downward trend during three developmental stages in ‘Yingshuang’ (Fig. 5B,D). Additionally, *CsDHAR2* and *CsGR* exhibited an upward trend of in ‘Huangjinya’ (Fig. 5B,E). *CsAO* increased prominently at stage 3, which was nearly 16-fold higher than that at stage 1 in ‘Anjibaicha’ (Fig. 5C). The expression level of *CsAPX* decreased slightly at stage 2 as compared with that at stage 1, and remarkably elevated at stage 3 in ‘Huangjinya’ (Fig. 5F).

### Expression profiles of genes involved in AsA biosynthesis and recycling in four tea plant cultivars

**Figure 6:**
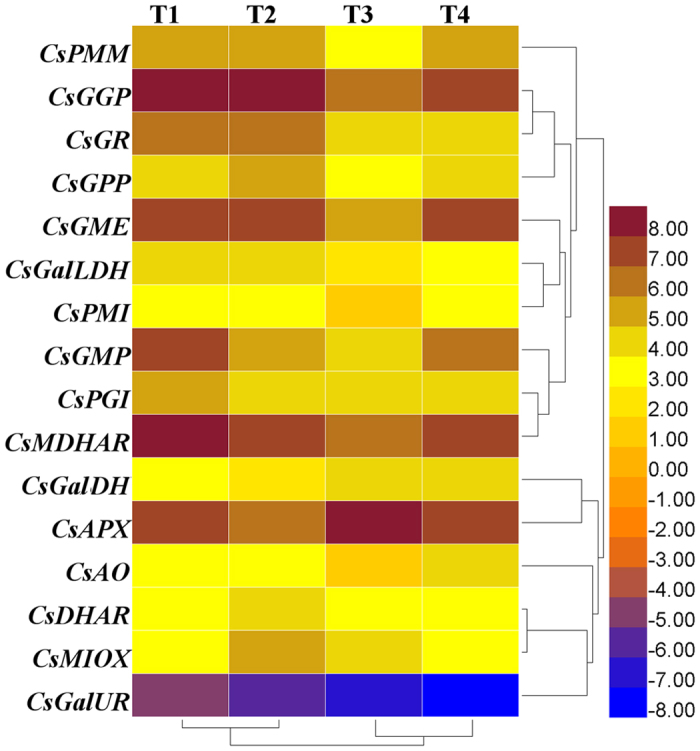
Heatmap of the relative expression level of genes involved in AsA biosynthesis and recycling in tea plant. T1 was ‘Yunnanshilixiang’, T2 was ‘Changwansanhao’, T3 was ‘Ruchengmaoyecha’, and T4 was ‘Anjibaicha’.

RNA sequencing (RNA-seq) data was extracted from transcriptome database in the four tea plant cultivars (Tea_T1, Tea_T2, Tea_T3, and Tea_T4)^41^. These tea plants were grown under non-stress conditions. The expression levels of genes involved in AsA biosynthesis and recycling were analyzed in another four other tea plants using RNA-seq data. RPKM values (Reads per kilobase per million mapped reads) were used to analyze the transcript levels of 18 genes, and a heatmap was obtained using HemI software (version1.0; http://hemi.biocuckoo.org/faq.php)^42^ (Fig. 6). *CsAPX* was expressed at the highest level (RPKM > 409) in Tea_T3. *CsGGP* showed a similar expression pattern in Tea_T1, Tea_T2, and Tea_T4. Both *CsPMI* and *CsAO* showed relatively low expression levels (RPKM > 2) in Tea_T3. *CsMDHAR* was highly expressed (RPKM > 291) in Tea_T1. In addition, *CsGMP* and *CsGME*, which participate in the AsA biosynthesis pathway were highly expressed in Tea_T1, Tea_T2, and Tea_T4. *CsMDHAR* and *CsAPX* were highly expressed in Tea_T1 and Tea_T4, whereas *CsGalUR* showed relatively low expression levels in the four tea plant cultivars.

## Discussion

### Different enzymes play various roles in AsA accumulation

Various enzymes are involved in ascorbic biosynthesis in higher plants. MIOX is a crucial enzyme in the *myo*-inositol pathway, but a number of scientific studies observed that the AsA content is insignificantly affected by MIOX. The AsA content did not increase in *Miox* overexpression lines compared with that in the wild type in *Arabidopsi*s^43^. Similarly, the levels of AsA content slightly changed in the *OsMIOX*-overexpressing transgenic rice lines compared with wild type^44^. GalUR is a pivotal enzyme in the d-galacturonate pathway. The expression level of the *VvGalUR* gene was correlated with the AsA content level during grape ripening^45^. By contrast, the AsA content showed no obvious relation to *GalUR* expression in kiwifruit^46^. The expression level of *CsGalUR* was negatively correlated with AsA accumulation from stage 1 to stage 3 in ‘Huangjinya’ and ‘Anjibaicha’. Whereas, the expression level of *CsGalUR* was positively correlated with AsA accumulation from stage 1 to stage 3 in ‘Yingshuang’. In addition, the expression level of *CsMIOX* was negatively correlated with AsA accumulation from stage 1 to stage 3 in ‘Huangjinya’. The expression level of *CsMIOX* was positively correlated with AsA accumulation from stage 1 to stage 3 in ‘Yingshuang’. Therefore, the *CsMIOX* and *CsGalUR* might play potential different roles in AsA accumulation in different tea plants.

### AsA accumulation and expression levels of genes involved in the biosynthesis pathway of AsA in tea plant leaves

The expression levels of genes involved in the AsA biosynthesis pathway varied in different tea plant leaves. A correlation was noted between the AsA contents and expression levels of genes involved in AsA biosynthesis in tea plant leaves. The highest expression level of *CsGGP* was correlated with the lowest content of AsA in each tea plant cultivar (Figs 2 and 4G). This finding indicated that *CsGGP* played a regulatory role between expression levels and AsA accumulation. Previous reports noted that *GGP* may function as a regulatory factor^47^. A positive correlation was found between *CsGPP* expression level and AsA accumulation from stage 1 to stage 3 in ‘Yingshuang’, whereas a negative correlation was observed from stage 1 to stage 3 ‘Huangjinya’ and ‘Anjibaicha’ (Figs 2 and 4H). In kiwi, Li *et al*. has also demonstrated that a positive correlation was found between *GPP* expression and AsA accumulation from 0 to 60 days after anthesis. Meanwhile, a negative correlation was found from 60 to 75 days after anthesis^48^. In AsA accumulation, l-galactose-1-P phosphatase (GPP) could use myo-inositol-1-phosphate as a substrate^49^. This finding suggested that *CsGPP* might be a critical regulatory factor in AsA content levels of leaves of three tea plant cultivars. *GPP* has been reported as an essential enzyme for AsA accumulation in tomato fruit^50^. In addition, *GME* and *GGP* shared a crucial role in controlling l-ascorbate biosynthesis in tomato, and *GME* and *GGP* transcripts were co-regulated^20^. This finding was consistent with our results in ‘Anjibaicha’ (Fig. 4F and G). Previous studies showed that overexpressed *GGP* gene from kiwifruit in *Arabidopsis* resulted in a five-fold increase in ascorbate levels; co-expressing the *GME* and *GGP* genes demonstrated a seven-fold increase in ascorbate levels^46^. The expression profile of *CsPMI* was positively correlated with *CsGR* from stage 1 to stage 3 in ‘Yingshuang’ and ‘Anjibaicha’ (Figs 4C and 5E), thereby suggesting a relationship of coordination and cooperation in AsA biosynthesis and recycling.

### AsA accumulation and expression levels of genes involved in the AsA recycling pathway

The recycling pathway of AsA is complicated in higher plants. According to theory of AsA-GSH (ascorbate and glutathione) metabolism^51^. AsA is first oxidized to form mono-dehydroascorbate by APX and AO and then regenerated by mono-dehydroascorbate (MDHA), which is extremely unstable^52^. Subsequently, DHA regenerates AsA under the action of DHAR and GSH^53^. Recent research showed that *MDHAR* negatively regulates ascorbate levels in tomato^54^. Expression of *MDHAR* was positively correlated with the AsA content during stage 2 to stage 4 in the radish root flesh, whereas the expression of *MDHAR* was negatively correlated with the AsA content from stage 1 to stage 2 in the radish root skin^55^. *MDHAR* was positively correlated with AsA content from stage 1 to stage 2, whereas it was negatively correlated with AsA content from stage 2 to stage 3 in the development of carrot root^17^. In the present study, *CsMDHAR* was negatively correlated with the AsA content from stage 1 to stage 2 in ‘Yingshuang’ and ‘Huangjinya’, whereas *CsMDHAR* was positively correlated with the AsA content from stage 1 to stage 3 in ‘Anjibaicha’ (Fig. 5D). These combined findings indicated that *CsMDHAR* might play a regulatory role in AsA content in ‘Anjibaicha’, ‘Huangjinya’, and ‘Yingshuang’. The results might provide more insights into improving the ascorbate levels and investigating molecular mechanisms in plant leaves.

### Potential pathway for AsA biosynthesis and recycling in tea plant

**Figure 7:**
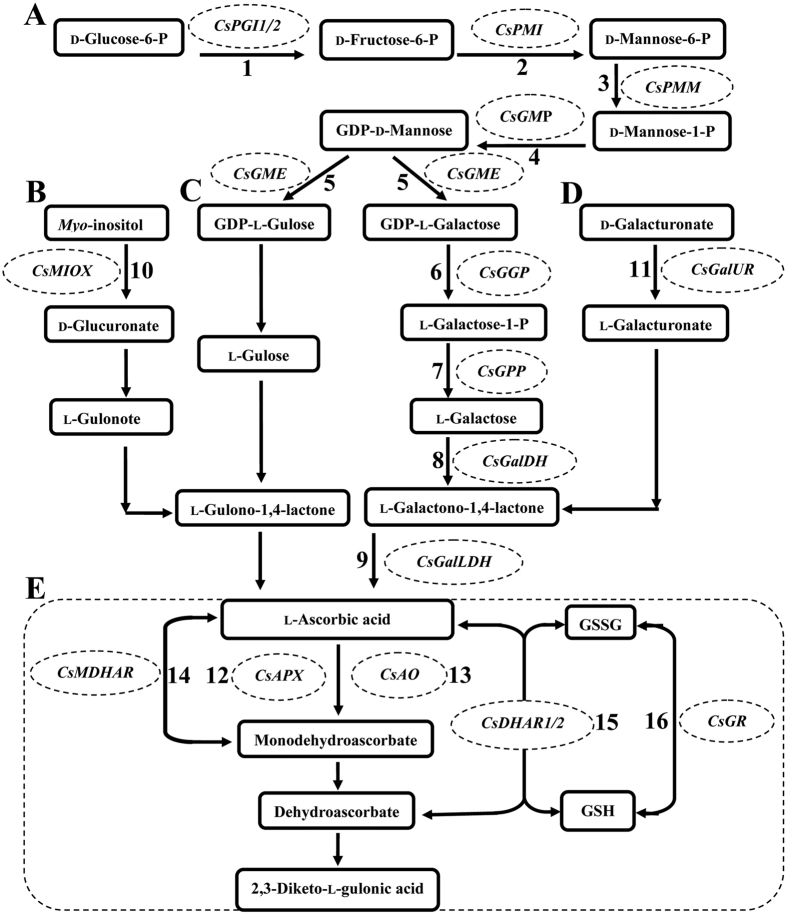
AsA biosynthetic and AsA recycling routes in tea plant leaves. (**A**) _L_-galactose (_L_-Gal), (**B**) *myo*-inositol, (**C**)l-gulose, (**D**) _D_-galacturonate and recycling pathway (**E**)). 1, phosphoglucose isomerase (PGI); 2, phosphomannose isomerase (PMI); 3, phosphomannose mutase (PMM); 4, GDP-d-mannose pyrophosphorylase (GMP); 5, GDP-d-mannose-3′,5′-epimerase (GME); 6, GDP-l-galactose phosphorylase (GGP); 7, l-galactose-1-P phosphatase (GPP); 8, l-galactose dehydrogenase (GalDH); 9, l-galactono-1,4-lactone dehydrogenase (GalLDH); 10, *myo*-inositol oxygenase (MIOX); 11, _D_-galacturonate reductase (GalUR); 12, ascorbate peroxidase (APX); 13, ascorbate oxidase (AO); 14, monodehydroascorbate reductase (MDHAR); 15, dehydroascorbate reductase (DHAR); and 16, glutathione reductase (GR).

Considerable evidence revealed that the l-Gal pathway was the major AsA biosynthesis pathway in several plant species. However, the network of this pathway is quite complicated. The l-gulose pathway, d-galacturonate pathway, and *myo*-inositol pathway also play a role in the regulation of AsA biosynthesis in several plants. Based on previous results about the AsA recycling pathways, the potential pathways of AsA biosynthesis and recycling during the development of tea plant leaves were established. Twelve genes involved in the AsA biosynthesis pathway and six genes related to the AsA recycling pathway were identified (Fig. 7).

## Conclusion

The AsA contents in leaves at three developmental stages of three tea plant cultivars were measured. By analyzing the expression levels of 18 genes, which were involved in the AsA biosynthesis and recycling pathways. The pathways of AsA metabolism were evaluated and predicted. The results indicated that the l-Gal pathway might be the major biosynthetic route for regulating the AsA content of tea plant leaves. Our findings suggested that the AsA biosynthesis and recycling pathways might be controlled by multigene regulation during the development of tea plant leaves. The results also demonstrated that AsA contents were intimately linked to gene expression. Moreover, the AsA biosynthesis and recycling pathways were confirmed. Further studies can explore the possibility to increase the AsA content *via* metabolic engineering and transgenic engineering in tea plant leaves.

## Materials and Methods

### Plant materials

The plant materials were two-year-old cutting tea plant seedlings called ‘Anjibaicha’, ‘Yingshuang’, and ‘Huangjinya’. They were planted in a growth chamber at 25 °C at the Tea Science Research Institute, College of Horticulture, Nanjing Agriculture University (Nanjing, China). The tea plants were grown in acidic soil (pH 5.6) with a relative humidity of 70 ± 10% and watered weekly. All of the three tea plant cultivars were collected from Zhejiang Province in China. The tea plant leaves were harvested, quickly frozen in liquid nitrogen, and stored at −80 °C for RNA extraction.

### AsA determination by RP-HPLC

AsA content levels were determined according to the method described by Guo and his colleagues^56^. In brief, 200 mg of fresh samples was ground in a mortar and homogenized 4 mL of 1.0% (w/v) oxalic acid. The mixture was transferred to a 10 mL centrifuge tube and centrifuged at 10,000 rpm for 10 min. Sample analysis by RP-HPLC was performed using the Shimadzu LC-20A series (Shimadzu Co., Kyoto, Japan) with a Hedera ODS-2 C18 analytical column (250 mm × 4.6 mm i.d., 5 μm nominal particle size) at 254 nm. About 20 μL of filtrate was injected in RP-HPLC for AsA determination. Finally, the AsA content was quantified by external calibration and results were recorded as mg/100 g FW.

### RNA isolation

Samples from three tea plant cultivars at three developmental stages were separately harvested. Total RNA of the samples was isolated in accordance with the method of a commercial RNA extraction kit (Huayueyang, Beijing, China). The Nanodrop 2000 spectrophotometer (Thermo Scientific, Wilmington, DE) was used to measure the concentration of isolated RNA.

### cDNA synthesis

First-strand cDNA of tea plant leaves at three developmental stages was synthesized with the PrimeScript RT reagent kit (TaKaRa, Dalian, China). The cDNA was diluted 15 times for PCR amplification. To explore the AsA metabolic pathway in tea plants, we selected a total of 18 AsA-related genes and determined their expression levels^41^.

### Gene expression analysis by qRT-PCR

Twelve genes involved in AsA biosynthesis and six genes involved in AsA recycling were identified based on the tea plant transcriptome database. The sequences of these 18 genes were shown on the attached data sheet (Supplementary Table 1). Primer Premier 6.0 software was used to design 18 pairs primer sequences. The primer length was restricted to 20–30 bp for the qRT-PCR (quantitative real-time PCR) (Table 1). The primer sequences with the cDNA template were checked by PCR (Polymerase chain reaction). To ensure the efficiency of optimal polymerization, the amplification length for each gene was restricted to 100–200 bp. The reaction program of qRT-PCR was performed under the following conditions: 95 °C for 30 s, followed by 40 cycles at 95 °C for 5 s, and 55 °C for 25 s. The volume reaction was 20 μL, which contained 2 μL of diluted cDNA strand, 7.2 μL of deionized water, 10 μL of SYBR Premix *Ex Taq* (Tli RNaseH Plus; TaKaRa, Dalian, China), 0.4 μL of forward primer, and 0.4 μL of reverse primer. The mean values and standard deviation were calculated based on three independent biological replicates. *CsActin* was used as a reference gene to normalize the expression of related genes involved in AsA biosynthesis and recycling of tea plants^57^.

### Statistical analysis

Differences in gene expression levels were detected by Duncan’s multiple-range test at a 0.05 probability level.

## Additional Information

**How to cite this article:** Li, H. *et al*. Transcriptomic analysis of the biosynthesis, recycling, and distribution of ascorbic acid during leaf development in tea plant (*Camellia sinensis* (L.) O. Kuntze). *Sci. Rep.* **7**, 46212; doi: 10.1038/srep46212 (2017).

**Publisher's note:** Springer Nature remains neutral with regard to jurisdictional claims in published maps and institutional affiliations.

## Supplementary Material

Supplementary Tables

## Figures and Tables

**Table 1:** Primers sequences of the related genes and reference gene used for qRT-PCR.

Name	Forward primer(5′-3′)	Reverse primer (5′-3′)
*CsPMM*	CCACATTATTAGCTTCCTTCTCGTCAC	CCAACAACACCAACTGTAACAACCTT
*CsGGP*	ATCTTCCTTGTACCACAGTGTTATGCT	TGCCTCCTCGTAGTCCTTCTTCC
*CsGalUR*	GAGCAGCCTCTTGGAGAAGCAAT	ATCACGATGAGCATCAGAACACCAA
*CsMIOX*	GCGTCAATCACATCAACCAAACTTT	GCTCATCTCCACCTTGTCCACTT
*CsGME*	AACTACGGAGCATA CACCTATGAGAAC	CTAGCAATGTGCGAGGCAATGAATC
*CsGMP*	GAACTCGGTTGAGACCATTGACACTT	CCACTTCACTCACTCCAATAGCCTTG
*CsGPP*	GCTGCTGGTGCTGTGGTAGAAT	CTAGAAGTGACTGCTCCACCTTATCG
*CsGalLDH*	GGCGGCATTGTTCAGGTTGGT	GTCCACAGCGAGCAAGATAGAATAGTT
*CsGalDH*	GAGAGTGACTAGGAGCATTGATGAGAG	CCAAGCGGAAGTCCTGTAATACCAA
*CsPMI*	TCTGCGGTCAATATTCACTCAACTCAT	TGTTCCTTATCTGTCAACTGCCTCAC
*CsPGI 1*	CATTGTGAAGAGTCAGCAACCTGTGTA	CGATTGCCAGAGAAGGTCTTGTGAG
*CsPGI 2*	CGATGTCGTCAGTGGTAAGATTAAGC	TTATCTTGAGAGGCGGATTATCAGGAG
*CsAPX*	AGCAAGGTCACGAAGCCAACAAT	GCAACAACTCCAGCCAACTGATAGA
*CsAO*	CCAACACCACTCAAGCACTAACAATAC	GAGGATGATACGGCGGTGATGG
*CsDHAR1*	ATGATGGAACCGAGCAAGCATTACT	GACAAGTCCGCAGCAGATACTCTT
*CsDHAR2*	ACCCTCCTCTCTGCCATTCTCC	TTCATCCAGTGCCTTCAACTCATCAA
*CsGR*	ACCCTGATGGCTAATAAGAATGCTGAA	TAGTATGTGCCTTGCCGAGTAGAGT
*CsMDHAR*	GGCGGATCAAGTGTTGGAAGGGAG	ACGCTTGGGATTGTATTCGGCATTA
*CsActin*	GATTCCGTTGCCCTGAAGTCCT	CCTTGCTCATACGGTCTGCGATA
