# Supplementary material for: Transcriptomic analysis of the biosynthesis, recycling, and distribution of ascorbic acid during leaf development in tea plant (Camellia sinensis (L.) O. Kuntze)
Source: Sci Rep. 2017 Apr 10;7:46212. doi: 10.1038/srep46212 (PMC5385563; doi:10.1038/srep46212)
Supplement: Supplementary Tables [file srep46212-s1.doc]

**Supplementary Tables**

**Transcriptomic analysis** **of the biosynthesis, recycling, and distribution of ascorbic acid during leaf development in tea plant** **(*Camellia sinensis* (L.) O. Kuntze)**

Hui Li^1^, Wei Huang^2^, Guang-Long Wang^2^, Wen-Li Wang^1^, Xin Cui^1^, Jing Zhuang^1^*

1.Tea Science Research Institute, College of Horticulture, Nanjing Agricultural University, Nanjing 210095, China

2.State Key Laboratory of Crop Genetics and Germplasm Enhancement, College of Horticulture, Nanjing Agricultural University, Nanjing 210095, China

*Please address all correspondence to: J Zhuang ([zhuangjing@njau.edu.cn](mailto:zhuangjing@njau.edu.cn))

-------------

Dr. Jing Zhuang

Professor

Tea Science Research Institute

College of Horticulture

Nanjing Agricultural University

Nanjing 210095, China

Fax: 86 25 84395182

Email: [zhuangjing@njau.edu.cn](mailto:zhuangjing@njau.edu.cn)

Supplementary Table 1 Nucleotide sequences of genes involved in AsA biosynthesis and recycling pathways.

| Name | Sequences |
| --- | --- |
| *CsPMM* | ATGTTCCTCCGTTGCAGATCTGCATCCGTTTCTGCATTCACACTCTACTCCACATTATTAGCTTCCTTCTCGTCACCCAATTTGCCCCAGAAAATTTTGTATACTGAAATGGCAGCAAGGAAACCTGGATTAATTGCTTTGTTTGATGTTGATGGCACTCTTACCGCTCCCCGAAAGGTTGTTACTCCGGATATGCTGAAGTTCATGCAGGAACTTCGAAAGGTTGTTACAGTTGGTGTTGTTGGAGGGTCTGACCTTGTTAAGATATCAGAGCAGCTTGGGAACTCAGTTATAAATGACTATGACTACGTATTTGCGGAAAATGGTCTTGTGGCCTATAAGGATGGGAAGCTCATTGGGACCCAGAGCTTGAAATCATATCTTGGAGAAGAAAAACTTAAGGAATTTATTAATTTTACGTTGCATTACATTGCCGACGTGGATATTCCAATAAAGAGAGGAACATTTATAGAGTTCCGAAGCGGGATGCTCAATGTATCACCAATTGGCCGAAACTGCAGCCAGGAAGAACGGGACGAGTTTGAAAAGTATGACAAGGTTCTGAACATACGCCCTAAGATGGTGTCTGTGCTTCGCGAGAAATTTGCTCACCTTAACCTCACCTTTTCCATTGGGGGACAAATAAGTTTTGATGTTTTCCCTCAAGGTTGGGACAAGACTTATTGCTTGAGATATGTGGATGATTTTCATGAAATTCACTTCTTTGGAGACAAAACTTACAAGGGAGGAAATGACCATGAGATATATGAATCGGAGCGAACTATGGGGCACACAGTTACCAGCCCTGAGGATACTGTAAAGCAGTGTACAGCTCTCTTCCTAAGCAAGCAGGTTTAA |
| *CsGGP* | ATGATGTTGAGGATCAAGAGGGTTCCTACGGTTGTTTCGAATTACCAGAAGGAAGAGGCGGAGGAAGGTGCTCGCCGCCCCGGAGGTGGTTGCGGCCGGAATTGCCTCCAGAACTGTTGCATTCTAGGAGCAAAGCTACCTCTGTATGCTTTCAAGAGGGTGAACAAGATTGTCGGTGAAAAGGGTCTAATTGCCCATGACTACATAGAGCCTTCCATTGCTTTCTTGGATTCTCTTCTTCTTGGGGAGTGGGAGGATCGTATGCAGAGAGGGCTCTTTCGCTATGATGTCACCGCTTGCGAAACCAAGGTAATCCCGGGAGAGTATGGATTCATTGCACAGCTGAATGAGGGCAGACACCTTAAGAAGAGGCCAACTGAGTTTCGTGTTGATAAGGTCCTGCAGCCCTTTGATGGGAACAAATTCAACTTCACTAAAGTTGGACAGGAAGAGGTGCTTTTCCAGTTTGAAGCAAGTGATGAAGACAATGAAACCCAGTTCTTCCCAAACGCACCCATTGATGTTGTGAATTCTCCAAGTGTCGTGGCCATCAATGTTAGTCCTATTGAGTATGGTCATGTACTTTTGATCCCTTGGATTCTTGAATGCCTGCCTCAGAGGATCGACAGGGTGAGTTTCTTGCTTGCTCTTTACATGGCAGCAGAAGCTGGAAACCCGTACTTCAGATTGGGTTACAACAGCTTGGGTGCATTTGCTACAATCAACCACCTTCATTTCCAGGCTTATTACTTGGCTGTGCCCTTTCCAATTGAGAAAGCTCCGATTAGGGAGATAACTACTTTGAATGGTGGGGTGAAAATCTCTGAGCTGTTAAATTATCCAGTCAGAGGTCTTGTTTTTGAGGGTGGAAATACTCTGGAAGATTTGTCCAATGCTGTCTCGGATTCCTGCATTTGCCTTCAAGATAACAACGTACCTTACAATGTGCTAATCTCTGAGTCTGGGAAACGGATCTTCCTTGTACCACAGTGTTATGCTGAGAAACAGGCTCTAGGAGAAGTGAGTTCTGAGCTTCTGGACACCCAAGTGAACCCGGCAGTGTGGGAAATTAGCGGGCATATGGTTTTGAAGAGGAAGAAGGACTACGAGGAGGCATCTGACAAAAATGCTTGGAGACTCCTTGCTGAGGTCTCCCTTTCCGAGGAGAGGTTCCAAGAAGTAAGTGCTTTGATCTTTGAATCCATCACTTGCATTGATGATGGAAATGAAAGTGTTTCTCAGAGCTTCATGGGGGAGCCAAATGCCACACCTCAACCTCTTGAAGAAGTCGATGACCTCGATAAATCATCCTGCCATGCTATGGTGCCCGGGAAGCAAGAATGCCTGGTTCTGCACTGA |
| *CsGME* | ATGGGAAGCGCAGGTGAAACTAACTACGGAGCATACACCTATGAGAACCTCGAGAGGGAACCCTACTGGCCTTCGGAGAAGCTCCGCGTTTCCATAACCGGAGCAGGTGGATTCATTGCCTCGCACATTGCTAGGCGATTGAAGAGCGAGGGGCATTATATCATCGCTTCCGACTGGAAGAAAAATGAGCACATGACCGAGGACATGTTTTGTCATGAGTTTCATCTTGTTGATCTGAGGGTGATGGATAACTGTTTGAAAGTCACTACTGGTGTTGATCATGTCTTCAACCTTGCTGCTGACATGGGAGGCATGGGCTTCATTCAGTCCAATCACTCAGTCATTATGTATAACAACACAATGATCAGCTTCAACATGCTTGAAGCAGCAAGGGTCAATGGAGTTAAGAGGTTATTTTATGCTTCCAGCGCTTGTATTTACCCTGAATTTAAGCAGCTGGACACTAATGTGAGCTTGAAGGAGTCTGATGCTTGGCCTGCTGAGCCTCAAGATGCTTATGGCTTAGAGAAGCTCGCAACTGAGGAATTATGCAAGCACTACACCAAGGACTTTGGCATTGAGTGTAGGGTTGGACGGTTTCATAACATCTACGGACCTTTTGGAACGTGGAAAGGTGGGAGGGAGAAAGCCCCTGCTGCATTCTGTAGAAAGGCCCTTACCTCAACTGATAAATTTGAGATGTGGGGAGATGGGCTACAAACTCGATCTTTCACGTTCATTGATGAATGTGTCGAAGGTGTCCTTAGATTGACAAAATCAGACTTCAGAGAACCAGTGAATATTGGAAGCGATGAAATGGTTAGCATGAATGAGATGGCTGAGATTGTTCTGAGCTTCGAGAACAAGAAGCTGCCTATCCATCACATTCCCGGCCCAGAGGGCGTGCGCGGTCGAAACTCAGACAACACTCTGATTAAGGAGAAGCTTGGTTGGGCCCCTACTATGAGATTGAAGGACGGGCTGAGAATCACATACTTTTGGATCAAGGAGCAAATTGAGAAAGAGAAGGCTAAAGGCATCAATCTGTCTACTTATGGGTCGTCAAAAATTGTGGGAACGCAAGCTCCAGTCCAGTTGGGCTCTCTTCGTGCTGCTGATGGCAAAGAATGA |
| *CsGMP* | ATGAAGGCTCTCATTCTAGTTGGTGGTTTTGGAACTCGGTTGAGACCATTGACACTTAGTGTCCCAAAGCCTCTTGTCGATTTTGCTAACAAACCTATGATCCTGCATCAGATTGAGGCTCTCAAGGCTATTGGAGTGAGTGAAGTGGTTCTAGCTATCAATTACCAACCAGAGGTGATGTTGAACTTCTTAAAGGACTTTGAGGCTAAACTTGGAATCAAAATCACGTGCTCACAAGAGACTGAGCCACTTGGTACTGCAGGTCCTCTGGCTCTGGCTAGGGACAAACTGATAGATGATTCTGGTGAGCCATTTTTTGTACTTAACAGTGATGTTATCAGTGAATACCCCCTCAAAGAGATGATCGAATTCCACAAATCCCATGGAGGCGAGGCTTCGATAATGGTAACCAAGGTTGACGAGCCATCAAAATATGGTGTGGTGGTTATGGAAGAATCAACTGGCCAAGTTGAGAGATTTGTAGAAAAACCTAAATTATTTGTGGGTAACAAGATCAATGCTGGAATATACTTGCTGAACCCATCTGTTCTTGATCGAATTGAACTGAGGCCCACCTCAATTGAAAAAGAGGTCTTCCCAAAAATTGCAGCCCAGAAACAACTTTACGGCATGGTTTTACCAGGCTTTTGGATGGACATTGGACAGCCTAGGGACTACATTACTGGCCTGAGACTTTACCTTGACTCCTTGAGAAAGAAATCTCCATCTAAATTGTCTACTGGACCTCATATTGTAGGAAATGTTCTGGTTGATGAGACCTCGAAAATTGGCGAAGGATGTTTAATTGGTCCTGACGTTGCAATCGGCCCAGGCTGTGTGGTTGAGGCAGGAGTTAGACTCTCTCGCTGCACCGTAATGCGTGGGGTCCGCATCAAGAAACACGCGTGCATTTCAAGTAGCATCATTGGGTGGCACTCAACTGTTGGGCAGTGGGCTCGCGTGGAGAACATGACAATTCTTGGAGAGGATGTTCACGTCTGCGATGAGATTTACAGCAATGGGGGCGTAGTTTTGCCACACAAAGAGATCAAATCGAGCATTTTGAAGCCAGAGATAGTTATGTGA |
| *CsGPP* | ATGGGAAGGTGTTTAGTCTCGTCCACACTCACTCCCCTGCGATTTTCTCAAATACGCAGATCAATTTCACTATTTAATCTCCCAAAACTCACTCTCCCATCTAGTTCTTTTGCAATTCCTCATCTGCGAAGAGGTTTCCGAGATGGGTTTTGCAAGACTCTGTCGTTCAATTCTATGCCCACAAGAACATTGTGCACCAAAGCTGTGTTATCTGAAATTCCCAATCAGAAAAAGTACTCAAAAGTTGCTTCTGAATCAACTGGCCCCATATCATCCAATCAGCTCCTCGGTGTGGTTGAAACTGCTGCTAAAACTGGTGCTGAGGTTGTGATGGACGCTGTGAAGAAGCCTCAAAATATTGTCTATAAAGGACTCACTGATTTGGTGACAGACACAGATAAAATGAGTGAGGTTGCTATTCTAGAAGTTGTAACGAAGAACTTCAAAGATCACCTTATTCTTGGGGAAGAGGGAGGGCTTATTGGCAATTCATCTTCTGATTATCTTTGGTGCATAGATCCCTTAGATGGGACAACAAATTTTGCACATTGCTACCCTAGCTTTGCAGTCTCTGTGGGAGTTCTGTATAAAGGAAAGCCGGCTGCTGGTGCTGTGGTAGAATTTGTTGGAGGCCCCATGTGTTGGAATACACGCACATTTTCTGCAGCTGCTGGTGGCGGTGCTTTTTGTAATGGGCAAAAGATTCAAGTGAGTCATACCGATAAGGTGGAGCAGTCACTTCTAGTTACTGGATTTGGGTATGAACATGACGATGCATGGAGTACCAACATAGAGTTATTCAAAGAATTTACTGATGTCAGCCGGGGCGTGAGAAGGCTTGGTGCTGCTGCAGTGGATATGTGCCATGTAGCTTTGGGAATTGTAGAAGCATATTGGGAATATCGTCTAAAACCATGGGATATGGCTGCTGGTGTTTTGATAGTTGAAGAAGCTGGTGGGGTAGTTTCTTGCATGGATGGTGGAAAATATAGTGTATTTGATAGATCAGTCTTGGTATCCAATGGTGTGCTGCATGACAAGCTTCTGGAGAGAATTGGCCCTCCAACGGATAAACTGAAGAACAAAGGCATTGATTTTTCATTGTGGTTTAAGCCAGAAAACTATCACACAGATTGTTGA |
| *CsGalLDH* | ATGGTCAATTTGGCTCTGTTGGATAGGGTTTTGGAGGTGGATAAGGAGAAGAAGACGGTTAGGGTTGAGGCGGGGATTCGTGTTCAGCAGCTTGTTGATGGGATCAAAGATTATGGACTCACTTTGCAGAATTTCGCTTCCATTAGGGAACAGCAGATTGGCGGCATTGTTCAGGTTGGTGCACATGGCACTGGTGCTAGATTGCCTCCTATTGATGAGCAGGTTGTCAGCATGAAACTGGTTACTCCTGCCAAGGGGACAATTGAGGTTTCAAAAGAGAAAGATCCAGAACTATTCTATCTTGCTCGCTGTGGACTTGGGGCCCTTGGCGTAGTTGCAGAAGTAACTCTTCAATGTGTTGAGAGACAAGAGCTCGTGGAGCATACATTTGTCTCAAATACGGAAGAGATAAAGAAAAATCACAAGAAGTTTCTATCTGAGAACAAGCATGTCAAGTATCTTTATATACCATATACTGACACTGTTGTGGTTGTGAGATGCAACCCTGTTTCCAAATGGAAAGGTCCGCCCAAGTTTAAACCAAAATATAGTCATGATGAAGCTATGCAGAATGTTCGTGACCTCTACCAAGAGTCTCTTAAGAAGTACAGACGTGCAGTGACAACAACCGAATCTGTGGACAACAATGAGCAAGACATAAATGAGCTTTCATTTACTGAGCTAAGAGATAAACTACTTGCCCTTGATCCTCTCAACAAAAACCATATCATAAAGGTCAATCAAGCTGAGGCAGAGTTCTGGAGGAAGTCAGAGGGATACAGAGTAGGCTGGAGTGATGAAATTCTGGGCTTTGATTGTGGCGGCCAACAGTGGGTATCAGAGACCTGTTTTCCTGCTGGAACCTTATCAAAGCCCAGCATGAAAGATCTTGAATATATAGAAGAGCTGATGCAACTTATAGACAAGGAAGCGATACCCGCACCGGCTCCTATAGAACAGCGATGGACGGCACGCAGCAAGAGCCTCATGAGCCCGGCTTCAAGTACAGCAGATGATGATATTTTCTCATGGGTTGGTATAATTATGTATCTTCCCACAATGGATGCTCGTCAGAGGAAAGAAATAACAGATGAGTTCTTCCACTACAGGCATTTGAGCCAATCACAGTTATGGGATCGTTATTCTGCTTATGAACATTGGGCTAAGATTGAGGTTCCAAAGGACAAGGACGAGCTTGCAACTCTGCAAGCAAGGTTGAGGAAGCGTTTTCCAGTGGATGCATACAATAAAGCACGAAGGGAATTGGACCCTAATCATATCCTTTCCAATAACATGCTGGAGAAGCTGTTCCCACAGTCGGATATAATTTGA |
| *CsGalDH* | ATGGCAAACCTTGAGCTCCGATCACTCGGAAACACAGGCCTCAAGCTCAGTTCCGTTGGCTTCGGGGCCTCACCTCTCGGCAACGTCTTCGGGCCTGTCTCCGAGGACGACGCCTTCGCCTCTGTACGCGATGCCTTTCGCCTCGGCATCAATTTCTTCGACACCTCTCCGTATTATGGAGGAACAGTGTCTGAGAAAGTATTGGGCAAGGCACTGAAAGCTATGGGGATGCCGAGAAACGAGTACATTGTGTCGACAAAGTGTGGGAGGTATGTTGATGGCTTTGATTTCAGTGCCGAGAGAGTGACTAGGAGCATTGATGAGAGCTTGGATAGGTTGCAGCTTGACTATGTTGATATATTGCAATGCCATGATATTGAATTTGGGTCTCTTGATCAGATTGTGAATGAGACTATTCCTGCTCTTCAAAAACTAAAGGAAGCAGGGAAGATCCGGTTCATTGGTATTACAGGACTTCCGCTTGGGGTTTTTACTTATGTCCTTGATCGGGTGCCACCAGGCACAGTCGATGTGATTCTGTCATATTGCCACTATAGTATTAATGATTCAACTCTGGAAGATCTACTGCCTTACTTGAAGAGCAAGGGTGTGGGGGTGATCAGTGCTTCTCCTCTTTCAATGGGGCTTCTTACAGAGCGTGGCCCTCCGGAGTGGCACCCAGCTTTGCCTGAACTCAAGGCTGCATGCCAAGCGGCTGCTGCTTATTGTAAAGAGAATGGGAAAAATATATCGAAGTTAGCTATGCAGTACAGCTTGTCAAATAAAGATATTTCTTCTGTACTGGTTGGCATGAACTCCGTGAAACAGGTTGAAGAGAATGTGGCTGCTGCTAAAGAACTCGCCATGTTTGGGAAGGATGAGAAAGCTGTGTCAGAGATTGAAGAGATATTAAAACCGGTGATGAATCAGACATGGCCTAGCGGTATACAACAAAGTTGA |
| *CsPMI* | ATGTTGATGGAGTCTAATGGTTCGTCCAATCGGCGCAGGACCCTTCAGAGACTGAGATGTTCGGTCAAGAACTACGATTGGGGTCGAATTGGCTGCGAATCAAGGGTTTCGAGGTTGTTCTCGCGTAATTCGGGGCTCCACATTGAGGAAGGCAAGCCTTATGCTGAGTTTTGGATGGGCACTCACGAGTCTGGACCCTCTTTCGTGCTTGAGAATGGGGGTTTGAGTTTGAACTCGTGGATTGCCAAAAACCCCAATGTTCTTGGCGATAAGGTTGTTCAGAAGTGGGGCGTTAATCTTCCTTTCTTGTTCAAGGTACTTTCAGTTTCAAAGGCATTGTCGATACAGGCCCATCCTGATAAGGAATTTGCTGGGTTTCTGCACAAGACTCGGCCTGATGTCTTCAAGGATGATAATCACAAGCCTGAAATGGCTTTGGCACTAACAGAATTTGAGGCCCTCTGTGGGTTTATTAGTCTCAAGGAGCTTAAGGATGTTCTTCAAAATGTTCCTGAGATTGTAGAAGTGGTTGGCAGTGTATATGCAAACCAAGTGTTAACCATCAACCATGAAGATGGGGAGGAAAAAGTAAAATCAGTTCTGCGGTCAATATTCACTCAACTCATGTCTGCTAGCAAGGATGTGATTTCCAAAGCACTATCCAATTTGAAAAGTCGCCTCAACCAGGTGAGGCAGTTGACAGATAAGGAACAGCTAGTATTGCGCCTAGAAAAGCAGTATCCAGCTGATGTCGGTGTCCTAGCAGCTTTCCTCTTTAACTATGTGAAGCTTAAACCAGGTGAAGCATTGTATCTAGGGGCAAATGAACTCCATGCCTACTTACATGGTGAATGTATTGAATGCATGGCAACTTCAGACAATGTTGTGCGTGCTGGCCTAACTCCAAAGAGCCGGGATGCCCAAATTCTTTGTTCCATGCTCACGTACAAACAGGGTTTTCCTGAAATTCTACAAGGAGTCCCGTTAAATCCATACACCAGAAGGTACCTCCCTCCTTTCGATGAATTTGAAGTTGATCGCTGCATTCTTCCCCAGGGTGCATCAGTTGTTTTTCCAGCAGTCCCTGGCCCCTCTGTTTTTGTGATCATGGAGGGAGAAGGAACAATGCATGCGTCGTCCTTTGAAGATGTAGTTAGGGAGGGTGATGTTCTATTCACTCCTGCAAATACTGATATAAGCGTGAGAACAGCATCTGAATTACATCTATATAGAGCTGGTGTGAACAGTCGGTTCTTTCAGGCGTCTTGA |
| *CsPGI 1* | ATGGCTTCATCTGCCCTTATCTGTGACAAGGAGCAATGGAAGGACTTGAAGGCCCATGTTGACGACATTAAAAAGACGCATTTACGCGAATTGATGAGTGACTCTGAGCGATGCAAATCAATGATGGCTGAGTTTGATGGGATACTATTGGACTACTCAAGGCAGTGTGCCACTCTTGAAACAATGAATAAGCTATTCAAGTTGGCAGAGGCTGCGTGTCTTAAAGAAAAAATTAACAAGATGTTTAATGGGGAGCGGATAAACAGCACGGAGAACAGGTCTGTCCTTCATGTAGCTCTTCGTGCTCCAAGAGATGCAGTTATAAACAGCAATGGAAAGAATGTCGTCCCAGATGTTTGGAATGTTCTGGACAAGATCCGGGATTTTTCTGAGAGGGTGCGCAGTGGTTCTTGGGTTGGAGCAACAGGAAAAGCATTGACAAATGTTATCGCTATTGGTATAGGTGGCAGCTTCTTAGGTCCTCTTTTTGTGCATACCGCTCTTCAAACAGATCCAGAGGCTATTGAATGTGCAAGAGGACGCCATCTGCGCTTTCTTGCAAATGTTGATCCAATTGACGTTGCTAGAAATATTACTGGGTTGAACCCTGAAACTACATTAGTTGTGGTGGTTTCAAAAACTTTTACAACAGCTGAAACTATGTTGAATGCTCGGACACTTAGGGAATGGATCTCATCTGCTTTGGGGCCTGAGGCTGTTTCAAAGCATATGGTTGCTGTCAGTACTAATCTAACGCTTGTAGAGAAGTTTGGGATTGACCCTAAAAATGCTTTTGCATTCTGGGACTGGGTTGGAGGCCGATATAGTGTTTGCAGCGCTGTTGGAGTATTTCCTTTATCTCTCCAATATGGGTTCTTGGTTGTTGAGAAGTTCCTGAAGGGAGCTTCAAGCATTGATCAACACTTCTATTCAGCGCCTTTTGATCAAAACATTCCTGTGCTTTTAGGTTTGTTGAGCGTATGGAATGTCTCATTTCTTGGATATCCTGCAAGAGCCATTTTACCTTATTCTCAAGCCCTGGAGAAGCTTGCCCCTCATATTCAACAGGTTAGCATGGAGAGTAACGGGAAAGGGGTATCAATTGATGGTGTGGCTCTTCCCTATGAGGCTGGTGAAATCGATTTCGGTGAACCAGGAACAAATGGTCAGCATAGCTTTTACCAATTAATTCACCAGGGGCGAGTTATTCCTTGTGATTTTATTGGCATTGTGAAGAGTCAGCAACCTGTGTACCTGAAAGGTGAAGTGGTGAGTAACCACGATGAGCTCATGTCTAACTTTTTTGCACAGCCAGATGCCCTTGCTTATGGGAAGACCCCACAACAGTTGCAAAGTGAGAATGTCTCCCAGCATCTTATCCCTCACAAGACCTTCTCTGGCAATCGGCCTTCTCTCAGCCTTCTACTTCCATCGTTGAGTGCTTATAATGTCGGACAGTTGCTGGCATTCTATGAACACAGAATTGCTGTTGAAGGCTTCATCTGGGGTATCAACTCCTTTGACCAGTGGGGAGTTGAGTTAGGAAAGTCGCTGGCTTCCCAAGTGAGAAAGCAACTTCATGCATCTCGTAAGAAAGGAGAACCAGTTGAGGGCTTCAATTTCAGTACTACAACAATGCTAAACAGATATCTACAGGAAAGTTCGGATGTACCTCCTGATCCATCCACTCTTCTTCCTCACATGTAA |
| *CsPGI 2* | ATGGCTTCGATCTCTGGTCTCAGCTCTTCTTCATCCACACTCAAACCCGAGAAGTTCACACCAAAATCAACCCCCTCTTCTTTACCTTCAAGAGATTCGATTGCTTTCCCCAATCGATCGAAGTTCTTCGATCGAGCTTCAACTCTCTCTCCTCAGTCCGTAGCTCGAGACATCCCAGCGAGCTTGTCGAGTACCAACGATGGCTTATCGAAGGAGAAGAAGAAAGGGTTGTTGAAAAATCCGAGAGAGCTGTGGCGGAGATACGTTGACTGGCTCTATCAGCACAAGGATTTGGGGCTGTATTTGGATGTGAGTCGAATCGGATTCACGGACGAGTTCGTGGGGGAGATGGAGCCCAAGTTTCAGGCGGCTTTCAAAGCCATGGAAGAGCTTGAAAAGGGATCGATTGCGAATCCTGATGAGGGGCGAATGGTTGGGCATTATTGGCTTCGAAACCCTAAGCTTGCTCCGAAGTCGATCCTGAGGTTGCAGATTGAGAACACGCTTGAAGCTGTTCGCAAGTTCGCCGACGATGTCGTCAGTGGTAAGATTAAGCCCCCATCCTCTCCGGAGGGTCGCTTTACCCATGTACTTTCTGTTGGAATTGGAGGTTCTGCCCTTGGACCACAGTTTGTTGCAGAGGCACTGGCTCCTGATAATCCGCCTCTCAAGATAAGGTTCATTGACAATACAGATCCAGCTGGCATTGATCATCAGATTGCTCAGCTTGGTCCTGAATTGGCTTCTACGCTTGTAATAGTGATATCAAAGAGTGGAGGTACTCCGGAAACTAGAAATGGTTTATTGGAAGTACAGAAGGCTTTCCGTGATTCTGGCCTGGATTTTGCAAAACAGGGTGTTGCTATTACACAAGAGAATTCTTTATTAGACAACACTGCAAGAATTGAGGGTTGGGTAGCTAGATTTCCAATGTTTGACTGGGTGGGTGGTAGAACCTCTGAAATGTCTGCAGTTGGTCTGCTTCCTGCAGCGCTTCAGGGAATTGACATTAAAGAAATGCTTGCTGGTGCATCATTGATGGATGAGGCAAATAGGACCACTGTGGTGAGGAATAACCCTGCAGCATTGCTAGCTTTATGCTGGTATTGGGCTTCTGATGGAGTAGGATCCAAGGATATGGTTGTTCTTCCATACAAGGATAGCCTATTATTATTTAGTAGGTATTTACAACAGTTGGTCATGGAGTCACTTGGGAAGGAGTTTGACTTAGAAGGTAATCGGCTGTGGAGGAGTGCTCATAGAACTCCCAAGCAACCTAGATCTTTTGGATGA |
| *CsAO* | ATGGGTGGTTCATTTTTTAACAGAATCTTAGTACCTTCCTCTTCTGCTGACCCATGTTCAAGGGCTTGTAGGGTTGCCCTTCTGATTTGTTGTTGTTTGTCGGTTTTGGTCGAGTCGTCTCTGGGATCCAAAACCAGGCATTTCAAATGGGAAGTTGAGTACATGTACTGGTCTCCCGACTGTGTCGAGGGTGTGGTGTTGGGGATCAATGGGCAGTTTCCGGGGCCAACTATCCGCGCTCGAGCGGGAGACATTGTTAATGTTGAACTTAAAAACAAGCTTACTACTGAAGGAGTTGTCATTCACTGGCATGGTATCAGACAGCAAGGAACACCATGGGCTGATGGAACTGCATCCATTTCACAATGTGTTATTAACCCAGGAGAAACCTTTGTCTATAGATTCAAAGTTGAGAGGGCGGGAACATATTTTTACCATGGACACTATGGGATGCAAAGAGCAGCAGGGTTATATGGGTCATTGATAGTTGACGTGGCAAAGGGAGAGAAAGAGCCATTCCATTACGATGGAGAGTTTAACATACTATTGAGTGATTGGTGGCACGAAAGCGTTCATGAACAAGAGCTCGGCCTCTCTTCCAAGCCATTTCGTTGGATTGGTGAACCCCAGACTTTGCTGATGAATGGTAGAGGACAGTACAATTGTTCTCTAGCTGCGCATTATAGCAATTCTTCATCCAGTCAGTGCATGTTTAGGGGTAACGAACAGTGCGCACCCCAGATCCTCCGAGTGCGTCCAAACAAAATCTACAGGCTCAGGGTTACCAGCTCCACCTCACTCGCATCTCTCAACTTGCAAATTTCGAATCACAAGATGGTGTTGGTTGAAGCTGATGGAAACTACCTACAACCATTCGCGGTGGACGATATGGACATTTACTCCGGTGAGAGCTACTCTGTTCTATTCACTACCGATCAAAACCCTTCCAACAACTACTGGGTTTCAGTAAGTGTAAGAGGTCGAAAACCCAACACCACTCAAGCACTAACAATACTTAACTATCACAACACAACGTCTGCCTCAAAACCTCCACCATCACCGCCACCAGTCGCTCCTCTATGGAACAACTACACCCACAGCAAATTATTCACCAAAAAAGTTCTAGCCCTAATGGGAAACCCTAATCCACCTCCGACCACCCATCACCGCCGTATCATCCTCCTCAACACCCAAAACTACATCGATGGTTACACCAAATGGGCCATCAACAACATCTCTTTAGTCCTACCTGCCACGCCTTACTTGGGTTCGATAAAATACAATCTAAACAACGCTTTCGATCACAAAACCCCCCCGGCAAACTTCCCAAGCAACTATGATGTCATGAAACAAGCCCAAAACCCAAACTCAACGTACGGGAGTGGAGTCTACATGCTCAGTTTCAACACCACAGTAGATATAATCCTTCAAAATGCCAACGCATTGGCCGTGAACACGAGTGAAATCCACCCGTGGCACTTACACGGCCATGATTTTTGGGTTTTGGGATACGGAGAGGGAAAGTTTTCGAATGAGAATGATGAAAAAAGCTTTAATTTGAAAAACCCGCCTTACAGGAACACCGCGGTGGTGTTTCCATTCGGATGGACCGCTCTGAGGTTTGTGGCGAATAATCCAGGAGTGTGGGCTTTTCATTGTCATATAGAACCTCATTTGCATATGGGTATGGGGGTTGTGTTTGCTGAAGGTGTCCGACGGCTTGGGAAGATACCAAACGAGGTCTTAACTTGTGGGTTGACAGGGAAGATGTTCTTGAACAACAAGAATGATTGA |
| *CsAPX* | ATGGGGAAGTGCTATCCAACTGTGAGCGAAGAGTACAAGAAGGCTATTGACAAAGCCAAGAGGAAGCTCAGAGGCTTCATCGCTGAGAAGAACTGTGCTCCATTGATGCTCCGTCTCGCATGGCACTCTGCAGGTACTTATGATGTGAACAGCAAGACCGGAGGTCCGTTCGGGACAATGAGGCACAAGCTTGAGCAAGGTCACGAAGCCAACAATGGCCTTGAGATTGCTGTCGGGCTCTTGGAGCCTCTCAAGGAACAGTTCCCAATACTCTCTTACGGCGACTTCTATCAGTTGGCTGGAGTTGTTGCCGTTGAAATTACGGGAGGACCTGATGTTCCATTCCATCCAGGACGGGAGGACAAACCTGAGCCACCAATTGAAGGCCGTCTTCCTGATGCTACTAAGGGATGTGACCATTTGAGGGATGTGTTTGTTAAACACATGGGCCTCAGTGACAAAGACATTGTTGTTCTCTCTGGTGGCCATACACTGGGAAGGTGCCACAAGGAGCGTTCTGGATTTGATGGAGCCTGGACTACCAATCCACTCATCTTTGATAACACCTACTTCACAGAACTCCTCACTGGAGAGAAGGAAGGCCTTCTACAGCTGCAATCTGATAAGGCTCTTCTCGACGATCCTGCTTTCCGCCCTCTTGTTGAGAAATATGCTGCGGATGAGGATGCTTTCTTTGCAGATTATGCAGAAGCTCACATGAAACTCTCTGAACTGGGGTTTGCTGAAGCCTAA |
| *CsDHAR1* | ATGTCGACCGCAAAAATTCATCCATCGGCTTCCGCACTATCTACAACTATCAAACACCTCACCGGCACCCTCCAATTTTCTCGCACCTGCACCTTTCCCCCCAACGGTTTGGCCCATTCGAGGCGCACTACACATGCACTCAGGATCAGGAGGAGCCTCACTGTGTCTTCTTCCTCTGTCTCCGACCCTCTCGAAGTCTGCGTCAAAGCCTCTCTCACTACCCCCAACAGGCTCGGTGACTGCCCCTTCACGCAAAGGGTTTTGCTGACACTGGAGGAGAAGCATCTCCCATATGACTTGAAGTTGGTTGATTTTTCTAAAAAGCCAGAATGGTTCTTAAAAGTAAGTCCTGAGGGTAAAGTTCCAGTGATAAAGATTGATGAGAAGTGGATTGCTGATTCAGATGTCATCACCCAAGCACTAGAAGAAAAATTCCCTAATCCACCGTTGGGAACACCACCTGAGAAAGCTTCAGTTGGATCAAAGATCTTCTCCACCTTCATTGGTTTCCTTAAAAGCAAAGACCCCAATGATGGAACCGAGCAAGCATTACTCAGTGAGCTGGTTGCTTTCAATGATTATCTCAAAGAAAATGGACCTTTTGTGGATGGGAAAAGAGTATCTGCTGCGGACTTGTCCCTTGGACCAAAGCTGTATCATTTAGAGATTTCACTGGGGCATTATAAAAACTGGTCAGTTCCAGATTCACTTCCCTGTGTGAAGTCCTACATGAAGGCCATATTCTCCATGGAGTCATTTGTCAAAACACGAGCTTTGCCAGAGGATGTGATCGAGGGTTGGCGACCAAAAGTGATGGGTTAA |
| *CsDHAR2* | ATGGCTTTGGAGGTCTGTGCCAAGGCTGCTTCTGGTGCTCCTGATATTCTTGGAGACTGTCCTTTTACCCAAAGGGTGCTTCTGACTTTGGAGGAGAAGAAAATCCCATACAAGATACATCTGATCAATATCAGTGACAAACCCCAATGGTTTTTGGAAGCGAACCCAGAAGGGAAGGTGCCTGTGATAAAATTTGATGAAGAATGGATTTCTGACTCTGATGTTATTGTTAGCCATATTGAAGAAAAATTCCCAGACCCTCCTCTCTGCCATTCTCCCGAGGTCTCCTCTGTTGGGTCCAAGATATTCCCTTCTTTTGTCAAGTTCCTGAAGAGCAAGGACCCCAATGATGGGTCGGAGCAGGCTTTGCTTGATGAGTTGAAGGCACTGGATGAACATCTTCAGGCACATGGACCGTATATTAATGGGGAAAGCGTTTGCGCTGTTGATTTGGGTCTGGCACCAAAGCTGTACCATCTTGATGTGGCTCTTGGCCATTTCAAGGGCTGGAAAATCCCAGAAAGCTTGACTCATGTCCATAATTACATGAAGTTGCTCTTCTCTAAGGAGTGTTTTGAGAAGACCAAGGCTGCAAAAGAACATGTGGTTGCAGGATGGGCGCCAAAGGTCAATCCATGA |
| *CsGR* | ATGGCGACCTCTCTGAGCGCACCAAAGCTGAGCACAACCTTCTCTTCTTCCCCAACCCTACAAACCCTCCGTAGAACCCTTCCAATCTCTCTCTCTCATCCCTTTCTCTCTCCTCACTCTCCCTCCTCTCCTCTCTTTCTCTCTCCTCGCCGCCTCCGCCCCTCTTTCTCTCACCACCACCACCGTCGCTTCTCAGCTCGAGCGGAGTCCGATAATGGCGCCGAGCCTCGCCACTACGATTTCGACCTCTTCACTGTCGGCGCCGGCAGCGGCGGCGTTAGGGCTTCTCGTTTCGCCGCCAATTTCGGCGCTTCCGTTGCCGTCTGTGAGCTTCCTTTCGCCACCATATCTTCCGAAACTAGCGGAGGCGTTGGTGGAACGTGCGTGCTTCGTGGATGTGTACCAAAGAAACTACTTGTGTTCGCATCCAAATATTCTCACGAATTTGAAGAGAGTCATGGTTTTGGATGGAAATATGAAACTGAACCCAAGCATGATTGGAGCACCCTGATGGCTAATAAGAATGCTGAATTGCAGCGCCTTATTGGTATCTACAAGAACATTCTGAAAAATGCTGGTGTCACTTTGATTGAAGGGCGTGGAAAGATTGTGGACCCACACACAGTGGATGTAGATGGGAAACTCTACTCGGCAAGGCACATACTAGTTGCAGTTGGGGGACGCCCTTCCATTCCTGAAATTCCCGGAAGTGAATATGCAATAGATTCAGATGCAGCCCTTGATTTGCCTTCAAGACCTGAAAAAATCGCAATAGTTGGGGGAGGTTACATTGCACTTGAATTTGCTGGTATCTTTAATGGTTTGAGAAGTGACGTCCATGTATTCATACGACAGAAAAAGGTTTTGAGAGGTTTTGATGAGGAGGTCAGAGATTTTGTTGCAGAACAGATGTCTCTAAGAGGGATTGAATTTCACACAGAGGAGTCACCTCAGGCTATCATTAAATCGGCAGATGGTTCACTATCTTTGAAAACTAACAGAGGAACAGTTGAAGGTTTTTCTCATATTATGTTTGCAACAGGCCGCAAGCCTAATACAAAGAATTTGGGGTTGGAGAAGGTAGGGGTAAAAATGGCAAAGAATGGAGCAATAGAGGTTGATGAGTTCTCCTGTACATCAGTTCCATCCATTTGGGCTGTTGGAGATGTTACAGATAGGGTGAATTTAACTCCAGTTGCTTTAATGGAGGGAGGGGCATTGGCGAAAACTCTTTTTAGGAATGAACCAACAAAACCGGATCATAGGGCTATTCCATCTGCTGTGTTTTCCCAGCCACCTATTGGACAAGTCGGCCTTACGGAAGAACAGGCTGTAAATGAATATGGTGATGTTGACATATTCACAGCAAACTTGAGGCCCTTAAAGGCTACACTTTCTGGACTTCCAGACCGGATTTTCATGAAACTTATAGTATGTGCAAAGACAAACAAAGTCCTTGGGTTGCACATGTGTGGAGAGGATTCACCAGAAATTGTGCAGGGATTTGCAGTTGCTGTGAAAGCTGGCTTGACAAAGGCTGAATTGGATGCCACAGTGGGTATTCACCCTACTTCAGCTGAGGAGTTTGTCACTATGAGGACTCCTACAAGGAAGATTCGAAACGGCCCTCCACCGGAGGGGACGATGGACTCTGTGGTTAAAGCTGCAGCAGGGGTTTGA |
| *CsMDHAR* | ATGGCGGAGAAGACTTTCAAGTACGTGATTCTCGGCGGTGGCGTCTCTGCTGGTTATGCGGCTAGGGAATTTGCCAAACAAGGAGTTAAGCCAGGCGAGCTTGCAATTATTTCCAAAGAGGGGGTTGCTCCTTATGAACGTCCAGCACTCAGCAAGGCATACCTGTTTCCTGAGTCACCTGCAAGACTTCCAGGGTTTCATGTCTGTGTTGGAAGCGGGGGAGAGAGGCTGCTTCCGGAGTGGTATGCACAGAAAGGAATTGCATTGATCCTCAATACAGAAATAGTGAAAGCAGATCTTGCTACAAAGACTCTCGTTAGTGCTGCTGGAGAAACTTTCAATTATCACTTCTTGATAATCGCAACTGGTTCTTCAGTTATAAGGTTGACAGACTTTGGTGTACAAGGTGCTGATGCCAAAAACATCTACTATTTGAGAGAAATTGATGATGCTGATAAACTTGTAGAAGCAATTCAGGCGAAGAAAAATGGAAAGGTTGTGATTGTTGGAGGAGGATACATAGGTCTTGAGCTGAGTGCAGTGATGAAGTTAAACAATCTCGATGTCAACATGGTTTACCCAGAACCGTGGTGCATGCCTCGGCTTTTCACAGCAGGTATAGCTGCTTTCTACGAAGGTTATTATGCAAATAAGGGAATTAAAATTATTAAGGGAACTGTAGCTGTTGGGTTTACTGCTGATGCAAATGGAGAGGTAAAGGAAGTAAAACTTAAGGATGGTAGAGTGCTGGAAGCAGACATTGTTGTTGTTGGAGTTGGAGGTAGACCTCTTACCACCTTATTCAAAGGACAGGTTGAAGAGGAGAAAGGTGGAATTGAGACCGACAGTTTCTTCAAAACAAGTGTTCCACATGTATATGCCGTGGGGGATGTTGCTACTTTCCCGATGAAAATCTACAATGAAATGCGAAGGGTTGAACATGTTGACCATGCCCGTAAATCTGCTGAGCATGCTGTGAAGGCTATATTTGCAAGCACAGAAGGGAAGTCAATTGAGGAGTATGACTACCTTCCATTCTTCTATTCCCGTTCCTTCAATCTGTCATGGCAGTTCTATGGCGACAATGTTGGCGACACTGTTCTATTTGGAGACAACAGCCCAACATCTGAAAATCCCAAGTTTGGTTCATACTGGATCAAAGACGGGAAGGTAGTGGGTGCTTTTCTGGAGAGTGGGACTCCTGAAGAAAACAAGGCTATTGCCAAAGTTGCAAGGGTCCAACCCCCAGTTGAGAGTTTGGACCTATTGGCAAAGGATGGTCTCACCTTTGCCTGCAAGATT |
| *CsGalUR* | AGCTCCGTGAAACTTTACTTCATATTTCTTTCCTCAAGTCTAGCATTACTTTCGTTCCAAAAAAAAAGTCTGTGTTTTTGGCTACATGTTCAAGCAATTTCACCATCCCATAAATCCTGAAGAGATTTATATGGGCCAACATCTGATATGAACTCAACTCCAGGAAATCCTTTACGCTGCAGAATTTGATTTATCATCTCGGAGTCTTGTGCGCTTAGCTCCCAATCAAATATGTGAAGGTTCTCTTTGATCCTCTCCTCGTTAAAGCTCTTCACCAGAACACTCACTCCTTGCTCATAAACCCATCTAAGACAAACCTGGGCAACAGATTTTCCTCTAGCTTTTGCAATCTGTTTGAGTACCTCACATTCCATGACTTGGTTACTTCCCCAAAGTGTTCCTTTGGCACCCAAAGGAGAGTAAGCTGTGACATGAATTCCATTTTTCTCACAAAAATCTCTTAGCTTCTTCTGTTGCCAAAGTGGGTTCATCTCAACCTGGTTGACAGCTGGAGGGATCTTTGCAGTTGATAGTAATAGTTGGAGCTTCTTGCATGAGAAATTACTGACTCCAATGGATTTTGTGAGGCCAAGACTCTGACACTCCTCCATTGCTTCCCAAACAGACTTGAAATCCAAGGGAAGAAGCTCTTGCTTATTTACAGGCAACTCATATTTCCCTGGCTTCGAGCTCACTGGCCAGTGAACAAGATAGAGATCAAGATATTCCAATCCAAGATTCTTGAGTGACTTTTGTATTGCAGGAAGGACATGATCACGATGAGCATCAGAACACCAAAGCTTAGAGGTGATGAAGAGGTCTTGGCGAGATTGAATGAAGCCTAGGCGTATGGCATCAGCAATTGCTTCTCCAAGAGGCTGCTCTGATCGGTATAGAGCCGCGGAGTCGAAGTGTCGGTAACCAAATTTGATTGCTTGGAGGATGGATTGTTTCATGGTTTCAGAGGATAAAAATGGATAAACAGCTGTTCCAAACCCCAGAAGAGGAATTTGCTTATGATCAGTACCAGTTGAAGAACACAGTAACATATCTGGAGTGCTTTTCATCATTTTTCCTATGTTCTGGAA |
| *CsMIOX* | TCATTACTTATATCGCTCATATTGGCCGAATATTCTCTGTTGTCTTGTCCTGAATGATAA  AATATGGAATTGATAAGTCTTTTAATAGACTATTTGAACTTTAACCCTCTTCACAAATAC  CCCCATTATATTATTCCAAAAGAACCATAAATACATAATATTATTTCACGATGTAGTAGT  CAACATCGAAGCACACAAACAAAAACAAAAAATCAAAACAAAAACCCCTAAAAAAAGAAA  TGCATCTAAAAGTCTCCATCACCATTCACACGCTAAAATTATCAACCCATAGCTCCAAAT  TTTCACCATCTCAGCTTCTCAGGGAAGTACTTTTTAATGAGAGAGAGATAGTATGGCTTG  ACTTTTTCGACATCGATTCTGACTTTGCTCTTACTGTATAGGTCATACTTGTTAAATATT  TGGAGCCACTTTAGATTCTCAATGTCTTCTTCATTCATCAAGTGTTTGTATGCTCCTGAC  TTATGTAAAGCATAAAATGAGTGATATCTGATAATGAAAAGGCCCGCTGAAGGTAAAGTT  GTATGATTTTCCTTAGCCACCAAATACATGTAGTCATCATGGCCCCATGACATCATCACA  TTGTTGAGTCCACAACCCTCAGAATAAATTCCAAATTTGGTGTTGTAAGCTGGATTTTCA  TAGTCTGCATTTTCTTTGAAGTACTTGTGATGGACAATTGATTCGTCAAAAGCACAGCCA  ACTGGGAATGTGTCACCTACAACAGCCCATTGAGGAAGCTCCCCAAAGCTAGGAAGAAGA  AGAACCTTCCCAAGATCATGGATAAGGCCAGTCAAGTGAAGCCAATCTTCATTGGGATAG  TCTTTCCTAATAGCTTCGGCTGTCTGCAATAAGTGCTCAATTTGAGGTTCATCCAAATCA  GGGTCACTCTCATCCACAACATCATTTAGAAGCTCACAACATTCCCATATGCTCATCTCC  ACCTTGTCCACTTTCCCATACTTTTCTCTCATCTTCTTCACGAAGTCAAAAGTTTGGTTG  ATGTGATTGACGCGGTAAAAATCCTCTACCCCTTGTTGCCTGAGACTCTCTGCATCATAA  TCCCTAAAGTTTTGGCCAAATGAATTGATCTCTGGCACCACAAATCCACCATCCAACACC  AATTCATTTTCATTGTTTGGGATCTTCTGTTCCTCATGTTCCTCATGAGCCTCAACTCCA  AAGTCAGGTTGATCAATGAGGATAGTCATCCTGAATCAAAACCCAAATTCAAAAATCTCA  CAAACTCAACTAAAAAAGAGAGAAAATCAACAAGTCTTCTTCTATGGGGTGGTGACATTG  TTGTGGAGGATTTTTGGTATTTATAATGACCGGACAGTCCTTCCCTGAGAATTCAGAGCT  TCCGAGTCCTTCCCTGAAAACTTCTTTCTATCCTTCTTTGAAATTACTTATATTATATAC  ATACAATAACACATAGAAAGAAAAAAATAATACTAATAATATTAAAATTAATACTGTGAG  TGGATATAGACATATACATGGATACACACAAATAAAAGAGAGGTATTAGAGAGAG |
